# Spontaneous Resolution of Tractional Retinal Detachment in a Type II Diabetic Patient

**DOI:** 10.7759/cureus.38010

**Published:** 2023-04-23

**Authors:** Fahad A Kandari, Abdullah A Albahlal, Rahma A Algethami

**Affiliations:** 1 Ophthalmology, King Khaled Eye Specialist Hospital, Riyadh, SAU; 2 Ophthalmology, King Faisal Specialist Hospital & Research Center, Riyadh, SAU

**Keywords:** extramacular retinal detachment, crunch phenomena, pars plana vitrectomy, proliferative diabetic retinopathy, tractional retinal detachment

## Abstract

A 43 years old male with diabetes type II was under treatment for diabetic retinopathy with extramacular tractional retinal detachment (TRD) in the left eye OS. During the follow-up visit, the patient had a drop in vision from 20/25 to 20/60. The TRD was found to have progressed to involve the macula and was threatening the fovea; therefore, vitrectomy was thought to be inevitable. Meanwhile, the patient adopted exercise and tight glycemic control, and during the preoperative evaluation of three months duration, we observed resolution of traction and return of visual acuity to baseline (20/20). In conclusion, spontaneous resolution of TRD is extremely rare. If it occurs, the patient may be spared from undergoing a vitrectomy.

## Introduction

Tractional retinal detachments (TRD) are the second most common form of retinal detachment [1]. TRD is mostly associated with proliferative diabetic retinopathy (PDR) [2]. At this stage of diabetic retinopathy, vascular endothelial growth factor (VEGF) is released, encouraging the development of fibrovascular proliferation (FVP) along the retinal surface and into the vitreous. A TRD results from the contraction of the posterior vitreous membrane leading to the contraction of FVP. Subsequently, the retina is pulled up and detached from the retinal pigment epithelium (RPE). Once the PDR progresses to a TRD, it might need surgical intervention with pars plana vitrectomy (PPV) [1]. Lifestyle modification in people with pre-diabetes delayed the onset of type II diabetes and reduced the incidence of microvascular complications [3].

Moreover, strict glycemic control can delay the development and progression of retinopathy in diabetic patients [4,5]. To the best of our knowledge, in patients diagnosed with diabetic retinopathy, spontaneous resolution of TDR has not been reported yet. However, spontaneous regression of the new vessels was reported in some studies [6].

## Case presentation

**Figure 1:**
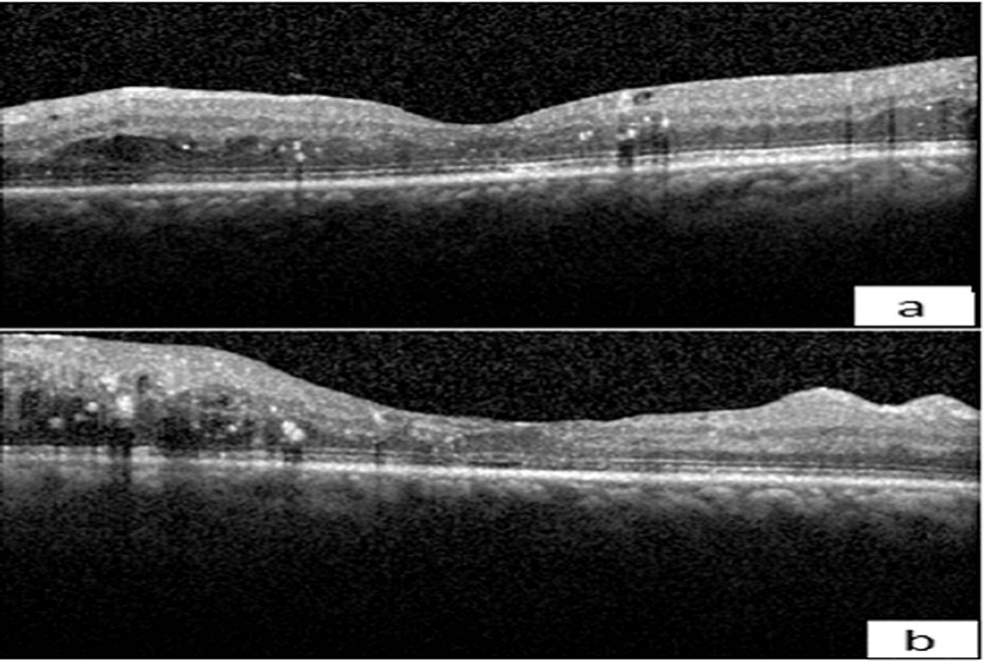
Initial visit horizontal spectral domain optical coherence tomography (SD-OCT) showing parafoveal cystoid macular edema. (a) Left eye (OS), (b) Right eye (OD).

**Figure 2:**
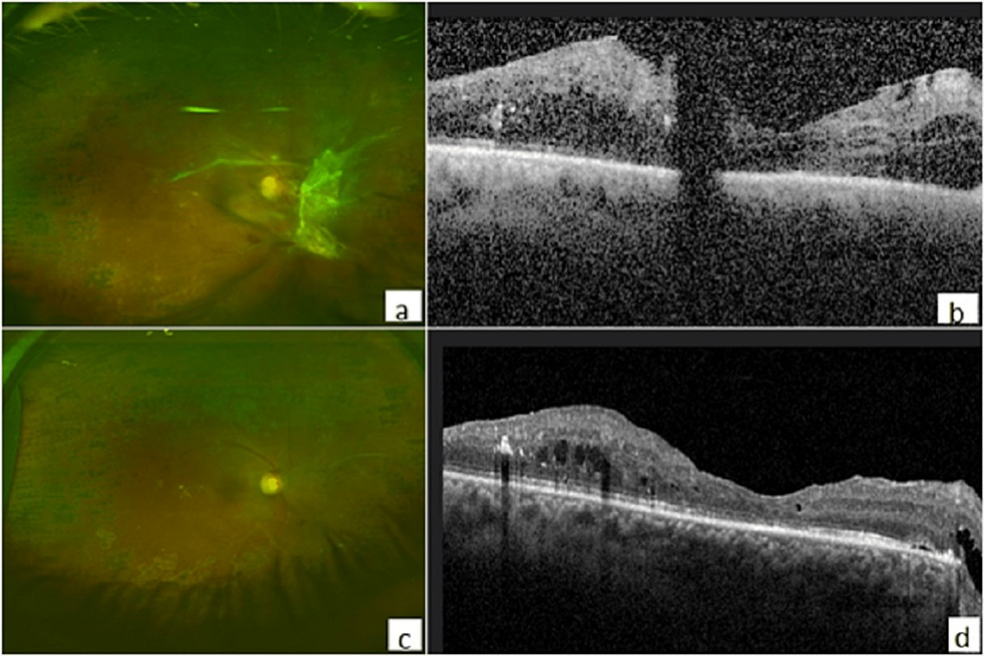
Right eye (OD). (a) Pre-operative fundus photo showing TRD threatening the macula. (b) Pre-operative spectral domain optical coherence tomography (SD-OCT). (c) Fundus photo post vitrectomy. (d) Post-operative spectral domain optical coherence tomography (SD-OCT). TRD: tractional retinal detachment

A 43-year-old male was known to have diabetes mellitus of 10 years duration, which was poorly controlled on oral hypoglycemic medication (HbA1c 9%). He presented with blurry vision in both eyes (OU) and has been diagnosed with PDR. The right eye (OD) showed a dispersive vitreous hemorrhage with progressive TRD threatening the macula. However, the left eye (OS) had an inferior vitreous hemorrhage with nasal extra macular TRD (Figure 1). The anterior segment was within the normal limit. Visual acuity on presentation was 20/300 in the right eye (OD) and 20/25 in the left eye (OS). Pan retinal photocoagulation (PRP) was performed in both eyes (OU) over multiple sessions with a total of 3500 shots in each eye; (power: 200-500 mW, duration: 200 ms, and spot size: 200 um). The patient received one intravitreal injection of bevacizumab (1.25 mg in 0.05 mL) in both eyes (OU). During the follow-up visit, the right eye (OD) showed tractional detachment progression involving the macula. Then, the right eye (OD) underwent PPV. Post-operatively, the right eye (OD) showed retinal flattening and involution of PDR with an improvement of visual acuity from 20/300 to 20/100 (Figure 2).

**Figure 3:**
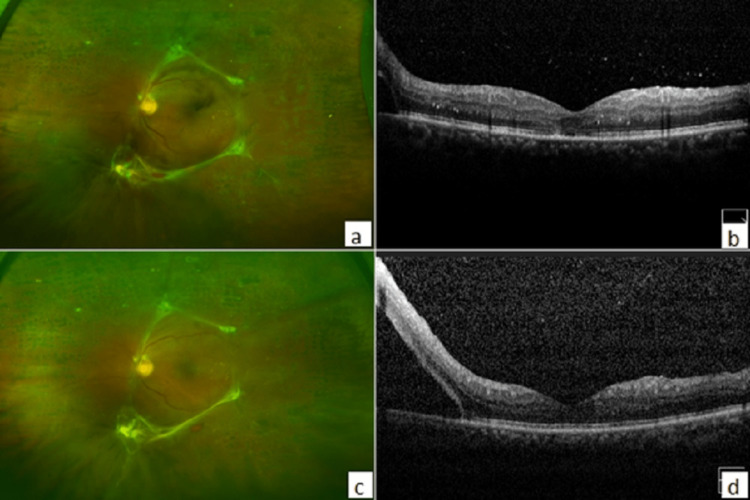
Left eye (OS). (a) Photo showing traction detachment on initial visit. (b) Horizontal spectral domain optical coherence tomography (SD-OCT) showing detachment away from macula on initial visit. (c) Photo showing the progression of detachment at six months follow up. (d) Horizontal spectral domain optical coherence tomography (SD-OCT) showing the progression of tractional detachment threatening the macula at six months follow-up.

**Figure 4:**
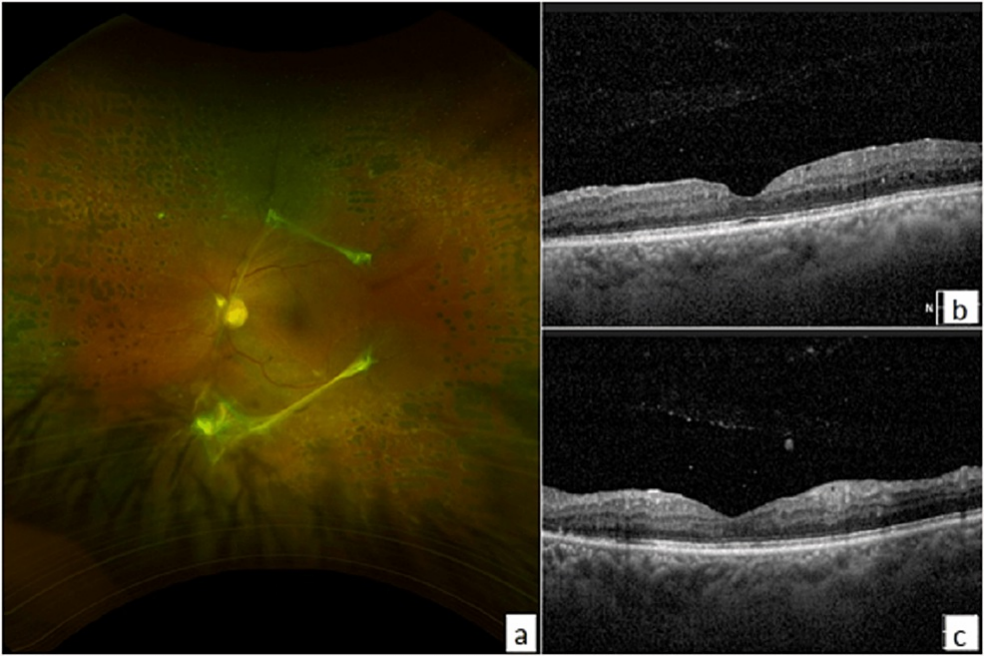
Left eye (OS). (a) Photos showing involution of diabetic retinopathy. (b) & (c) Horizontal spectral domain optical coherence tomography (SD-OCT) showing spontaneous resolution of TRD at two years follow-up. TRD: tractional retinal detachment

During the three months follow-up time, extramacular tractional detachment in the fellow eye (left eye OS) had also progressed to involve the inferior macula with significant parafoveal subretinal fluid and a drop in visual acuity from 20/25 to 20/60 (Figure 3). The patient was counseled about the need for urgent surgery in this eye after the right becomes stable. During this period, the patient adopted exercise and tight glycemic control (HbA1c improved to 6%). Surprisingly, on pre-operative evaluation, the patient was found to have improved to 20/20 vision in his left eye (OS) with the resolution of traction from the posterior pole and the resolution of the parafoveal subretinal fluid. The patient maintained the stability of visual acuity and clinical findings over two years of follow-up (Figure 4).

## Discussion

Diabetic retinopathy continues to be a leading cause of visual loss worldwide [7]. It is classified into non-proliferative diabetic retinopathy (NPDR) and PDR [8]. PDR is treated by ablating the ischemic retina using laser photocoagulation or injection of intravitreal anti-VEGF, while NPDR usually requires stress on glycemic control and observation [1,6]. Patients with neovascularization at the disc (NVD) have a poorer visual prognosis, a high incidence of fibrous proliferation, TRD, and vitreous hemorrhage [9].

Hypertension and nephropathy may worsen retinopathy [10,11]. The Diabetes Control, and Complications Trial (DCCT) established that intensive glycemic control in type I diabetes reduced the risk of development of retinopathy and slowed its progression in a group with mild retinopathy at baseline [4].

Several studies have confirmed the importance of physical activity in achieving glycemic control in diabetic patients. A meta-analysis demonstrated that simple walking reduces HbA1c by 0.5% [12]. However, several features of exercise that can reduce HbA1c in type II diabetes modalities of aerobic exercise alone or combined with resistance training showed significant reductions of 0.6% in HbA1c [13,14]. The exercise duration for more than 12 months and more than 150 minutes per week showed more reduction in HbA1c compared to those who do the exercise for a period of fewer than 12 months or less than 150 min per week [15,16]. Furthermore, frequent physical activity is associated with a lower incidence of severe diabetic retinopathy [17]. In our case, the patient adopted exercise and tight glycemic control with HbA1c improving to 6% over three months (originally 9%), which might have contributed to the favorable clinical course.

In treating high-risk PDR, combined intravitreal anti-VEGF and PRP may lead to regression of retinal NV and improvement in vision [18]. In our case, the patient received multiple sessions of PRP and one intravitreal injection of bevacizumab in both eyes OU during the follow-up period.

The incidence of severe visual loss decreases by about 50% when the retina PRP is performed before developing severe PDR‑related complications such as TRD and vitreous hemorrhage [19]. However, TRD involving or threatening the macula is a classic indication of vitrectomy, and a non-surgical approach is unlikely to resolve a TRD [20]. In our case, tractional detachment spontaneously resolved without surgical intervention, and vision returned to 20/20, which is unusual.

Our patient had reasonable glycemic control and regular exercise; this might have contributed to the involution of diabetic retinopathy. However, to have a resolution of tractional forces, it is required to have a disinsertion of at least one epicenter either surgically or spontaneously, as in our case. It is well known that PRP and anti-VEGF in diabetic patients might lead to anterior-posterior contraction of the posterior hyaloid (Crunch Phenomena) [21,22]. We hypothesize that this contraction occurred in our patient following PRP/anti-VEGF injection against a weak epicenter, leading to spontaneous disinsertion from the retina.

In a similar reported case, a patient had improved vision, in addition to spontaneous resolution of the tractional detachment without requiring surgical intervention, after he followed a ketogenic diet which led to significant weight loss and, subsequently, better control of blood sugar, HBA1c, and ocular condition [23]. Moreover, a case reported that spontaneous regression of NVD in a patient with type I diabetes was expected to be related to blood sugar control and weight loss as the patient underwent bypass surgery one year before the presentation [5]. Regarding the factors that lead to spontaneous regression of diabetic retinopathy, a previous study conducted on the Chinese population to assess the systemic factors related to diabetic retinopathy regression, results showed that diabetic retinopathy regression often occurred in patients with lower baseline HbA1c levels, shorter diabetes duration, and normal serum triglyceride levels [24].

## Conclusions

In summary, we believe the traction release may be attributed to the mechanical disinsertion of the weakly adherent epicenter that contributed to the detachment's tractional force. However, strict glycemic control and regular exercise may have helped in the PDR regression.
